# Prevention of 90-day inpatient detoxification readmission for opioid use disorder by a community-based life-changing individualized medically assisted evidence-based treatment (C.L.I.M.B.) program: A quasi-experimental study

**DOI:** 10.1371/journal.pone.0278208

**Published:** 2022-12-15

**Authors:** Zhehui Luo, Canopy Roychoudhury, William S. Pompos, James DiMaria, Cynthia M. Robinette, Purva H. Gore, Rohon Roychoudhury, William Beecroft

**Affiliations:** 1 Department of Epidemiology and Biostatistics, Michigan State University, East Lansing, Michigan, United States of America; 2 Health Care Value Business Analytics Services, Blue Cross Blue Shield of Michigan, Detroit, Michigan, United States of America; 3 Behavioral Health Strategy & Planning, Blue Cross Blue Shield of Michigan, Detroit, Michigan, United States of America; 4 College of Osteopathic Medicine, Michigan State University, East Lansing, Michigan, United States of America; Duke University Medical Center: Duke University Hospital, UNITED STATES

## Abstract

**Background:**

Evidence for community-based strategies to reduce inpatient detoxification readmission for opioid use disorder (OUD) is scant. A pilot program was designed to provide individualized structured treatment plans, including addressing prolonged withdrawal symptoms, family/systems assessment, and contingency management, to reduce readmission after the index inpatient detoxification.

**Methods:**

A non-randomized quasi-experimental design was used to compare the pilot facilities (treatment) and comparison facilities before and after the program started, i.e., a simple difference-in-differences (DID) strategy. Adults 18 years and older who met the Diagnostic and Statistical Manual of Mental Disorders version 5 criteria for OUD and had an inpatient detoxification admission at any OUD treatment facility in two study periods between 7/2016 and 3/2020 were included. Readmission for inpatient detoxification in 90-days after the index stay was the primary outcome, and partial hospitalization, intensive outpatient care, outpatient services, and medications for OUD were the secondary outcomes. Six statistical estimation methods were used to triangulate evidence and adjust for potential confounding factors between treatment and comparison groups.

**Results:**

A total of 2,320 unique patients in the pilot and comparison facilities with 2,443 index inpatient detoxification admissions in the pre- and post-periods were included. Compared with patients in comparison facilities, patients in the C.L.I.M.B. facilities had higher readmission in the pre-period (unadjusted readmission 17.0% vs. 10.6%), but similar rates in the post-period (12.3% vs. 10.6%) after the implementation of the pilot program. For 90-day readmission, all DID estimates were not statistically significant (adjusted estimates ranged from 6 to 9 percentage points difference favoring the C.L.I.M.B. program). There was no significant improvement in the secondary outcomes of utilizations in lower level of care and medications for OUD in C.L.I.M.B. facilities.

**Conclusions:**

We found a reduction in readmission in the pilot facilities between the two periods, but the results were not statistically significant compared with the comparison facilities and the utilization of lower level of care services remained low. Even though providers in the pilot OUD treatment facilities actively worked with health plans to standardize care for patients with OUD, more strategies are needed to improve treatment engagement and retention after an inpatient detoxification.

## Introduction

Almost 500,000 Americans died from an opioid-related overdose between 1999 and 2019 [1]. Although rates of opioid-related U.S. hospital discharges including detoxification services decreased from 31.6 to 27.4 per 100,000 in the general population between 1993 and 2016 [2], among individuals who received treatments for opioid use disorder (OUD) in the prior 12 months, the use of inpatient addiction treatment increased from 38% to 52% from 2004 to 2013 [3]. Rarely an inpatient detoxification admission results in a complete navigation of the inpatient treatment system [4]. Among inpatient detoxification patients during 2003–2011, only 13% received rehabilitation during inpatient care and up to 14% were discharges against medical advice [5]. Initiating medications for OUD (MOUD) during a hospital admission [6] or continued patient navigation service after discharge [7] have been found to reduce readmission in randomized controlled trials (RCTs). Methadone, naltrexone, and buprenorphine have been approved for the treatment of OUD, which are medications that can fully or partially function as an antagonist to the mu receptor in the nervous system [8]. However, tightly controlled trials in special settings are limited in generalizability. In real world settings, fewer than 20% to 30% of individuals in inpatient detoxification settings were offered MOUD [9, 10]. Because detoxification without further treatment only addresses physical dependence in the short term, relapse to opioid use and readmission are common [11]. It has been conjectured that because tolerance is reduced by detoxification, at the time of relapse the risk for overdose and death is high [12]. Evidence-based strategies–including increasing access to treatment and harm-reduction programs after the inpatient management of withdrawal–must be adapted and deployed to address the opioid epidemic and save lives [13, 14].

In the past few decades, OUD has been treated on an episodic basis with poor outcomes and high relapse rates [15–17]. Many patients and providers lament the mismanagement of OUD and unpreparedness of many treatment facilities to meet the special needs of patients with histories of unemployment, homelessness, and psychiatric comorbidities [18, 19]. To address these issues, we need to consider the chronic relapsing course of the disease and design strategies for relapse prevention as well as withdrawal management in the community [20].

Grounded in the community-based chronic care model, a pilot program (Community-based Life-changing Individualized Medically assisted evidence-Based treatment [C.L.I.M.B.]) was implemented on 5/1/2018 and 12/1/ 2018, by the Blue Care Network (BCN) and Blue Cross Blue Shield of Michigan (BCBSM), respectively. The program emphasized the chronicity of the illness and utilized a smart phone application (app) to facilitate management of withdrawal symptoms in outpatient services and prevent relapses. It was an alternative utilization management process that allowed more time to be spent in the residential/domiciliary portion of treatments while mid-level and outpatient services could be extended even further out. This paper evaluated the impact of the program on inpatient detoxification readmission in 90 days after the discharge. The research question is: If the C.L.I.M.B. program is rolled out to facilities like the pilot facilities, will it reduce 90-day readmission among OUD patients who were initially treated in an inpatient setting? We hypothesized that the pilot program patients would experience more reduction in 90-day readmission (primary outcome) and increase in non-inpatient services utilization and medication for OUD (secondary outcomes) compared with patients in other facilities.

## Methods

### Design

**Fig 1:**
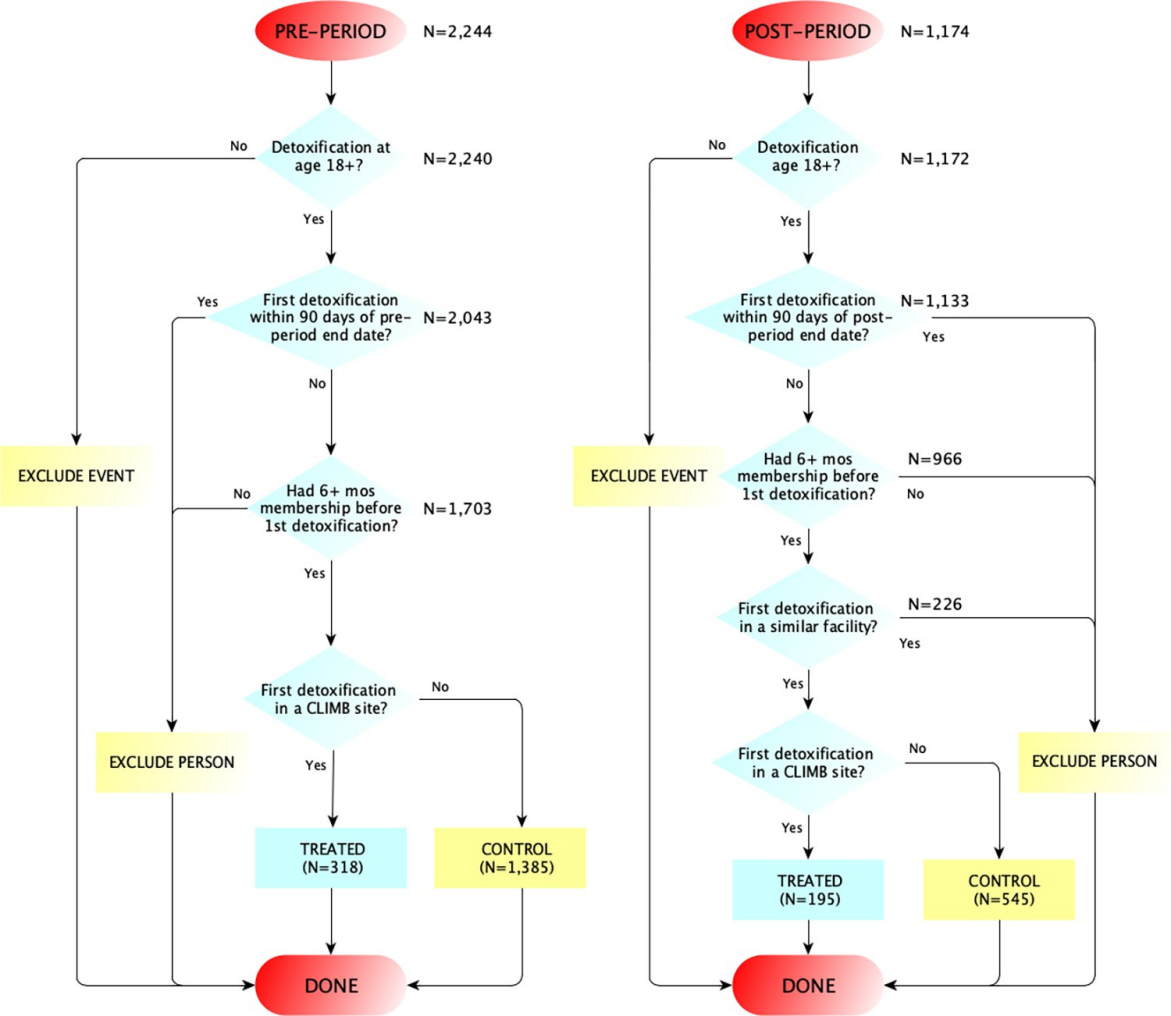
Patient inclusion and exclusion criteria and sample sizes in the pre- and post-periods.

Consistent with the quasi-experimental two-group pre-post design, the difference-in-differences (DID) method was used to ameliorate potential confounding bias [21, 22]. The underlying assumption of the DID method is that the change in readmission rates from pre- to post-period in comparison facilities is a good proxy of the counterfactual change in the pilot facilities had there been no pilot program (S1 Fig). The effect of interest is the average treatment effect on the treated which answers the question: for patients treated in the pilot facilities, was the program a cause for the change in readmission rate? On the probability scale, a DID method estimates the difference of risk differences (DRD); and on the odds ratio (OR) scale, a DID method estimates the ratio of ORs (ROR). The pre-and post-periods for the BCN (a health maintenance organization [HMO]) patients were 7/1/2016 to 4/30/2018 and 5/1/2018 to 8/31/2019; the corresponding periods for the BCBSM (a preferred provider organization [PPO]) patients were 2/1/2017 to 11/30/2018 and 12/1/2018 to 3/30/2020. In the post-period, another substance use treatment program implemented a program like C.L.I.M.B.; thus, to avoid contamination, patients whose index inpatient detoxification occurred at that site in the post-period were excluded (Fig 1). The pilot program was approved by the BCN and BCBSM medical directors, and the current evaluation was approved by the Institutional Review Board of Michigan State University as non-human subject research (STUDY00000846).

### Patients

Patients 18 years or older, who had a hospital-based detoxification inpatient stay for a diagnosis of OUD in any of the two periods were included in the study. To ensure data completeness, a patient had to be enrolled in the health plan for 6 or more months before the index inpatient detoxification to capture baseline comorbidity; and the index inpatient detoxification did not occur within 90-days of each period’s end date. Patients’ insurance membership file, medical (inpatient, outpatient, office-based encounters), and pharmacy claims from 1/1/2016 to 3/30/2020 were used to identify these patients.

### Intervention

Following ASAM guidelines [23, 24], the C.L.I.M.B. facilities, which are addiction treatment facilities affiliated with two hospitals, included services for the continuum of OUD cycle (S1 Fig), including detoxification, residential service, partial hospitalization/intensive outpatient service, outpatient service, MOUD, and a modified smartphone app, called A-CHESS, originated in 2011 at the University of Wisconsin Center for Health Enablement Support System (CHESS), which is a comprehensive tool based on the self-determination theory [25] to help patients with substance use disorders (SUDs) succeed in recovery (S1 Appendix).

Prior to the pilot program implementation, providers in the two facilities provided the same services as other OUD treatment facilities. During the implementation, they agreed to follow the C.L.I.M.B. codified protocol (detailed in the supplemental materials) with an emphasis on the master-treatment-plan development, family/system assessment, warm handoff, completion of tasks regardless of length of stay, and the use of A-CHESS. The protocol included planning for a suitable recovery environment and initiating MOUD if appropriate. Evidence-based pharmacotherapy options such as Buprenorphine and Naltrexone may be considered. These medications that in essence block or partially block the mu reception decrease the effect of opioids and give a neutral reward for use of the intoxicating substance [26].

Key features of A-CHESS [27] included:1) a “Help” button linked to the patient’s preapproved supporters, 2) positive and potentially distracting games, and audio-video relaxation recording; 3) cognitive behavioral therapy boosters; 4) functionality monitoring with self-assessment tools; 5) a global positioning system location tracker that will initiate a patient-defined action (e.g., contacting sober coach) when s/he approaches a high-risk location, and 6) just-in-time feedback via a counselor dashboard.

### Comparison group

All other OUD treatment facilities (could be a hospital or a standalone SUD treatment facility) in the U.S. that BCBSM and BCN members attended for inpatient detoxification in the study period constituted the comparison group. The usual care available at each facility varied and was expected to be representative of current practice in the field. Not all facilities covered the continua of all levels of care (LOCs).

### Main measures

#### Primary outcome

90-day inpatient detoxification readmission after an index inpatient detoxification discharge at any facility. A key purpose of the pilot was to reduce relapse and readmission by allowing patients a longer domiciliary stay if needed. Readmission was identified by the same method as the index inpatient detoxification: any inpatient stay with a diagnosis of F11.x or F11.xx using the International Classification of Diseases, 10^th^ version, Clinical Modification (ICD-10-CM) codes, and revenue codes 01x6 (x = 1, 2,3,4, or 5).

#### Secondary outcomes

Other ASAM LOCs, including intensive outpatient services, partial hospitalization services, domiciliary behavioral treatment, behavioral therapy, outpatient services, and MOUD. Because the first 30-day post discharge is crucial for treatment entry and engagement, we measured the receipt of these services between day 1 and day 30 after the discharge of the index detoxification. MOUD was identified using National Drug Codes in pharmacy claims and the Current Procedural Terminology codes; and revenue codes and/or procedure codes were used to find LOC 1.0–2.5 services (the list of these codes is available upon request).

#### Treatment groups

The National Provider Identifier codes for the two pilot facilities were used to identify patients in the pilot group. Patients in the other treatment facilities were the comparison except those in a facility that implemented a similar pilot program in the post period.

#### Comorbidity

The Agency for Healthcare Research and Quality Clinical Classification Software Refined version (v2021.2) [28] based on ICD-10-CM codes was used to find in medical claims comorbid conditions in the 6 months prior to the index inpatient detoxification in each period. Mood disorders, anxiety-, fear-, trauma- or stressor-related disorders [29], alcohol-, cannabis-, sedative-, stimulant- hallucinogen- or inhalant-related disorders [30], and suicidal ideation/attempt or intentional self-harm [31] frequently cooccur with OUD and may increase the complexities of disease management. Other non-SUD comorbid conditions, including neoplasms [32], endocrine, nutritional and metabolic diseases [33], and diseases of the nervous, circulatory, respiratory, digestive, musculoskeletal system, or genitourinary systems, are used to control for patients’ chronic conditions [34]. Emergency room visits [35] in the 6 months prior to the index inpatient detoxification in each period were identified using revenue codes 045x (x = 0–9).

#### Covariates

Patient’s age, sex, HMO or PPO plan types, and residential zip codes were extracted from health plan enrollment files. The 5-digit zip codes were linked to the census tracts using the U.S. Department of Housing and Urban Development zip code crosswalk files [36] where a census tract with the highest residential ratio was chosen when multiple tracts were within the same zip code [37]. Past research found that living in a disadvantaged neighborhood was associated with worse health conditions and increased healthcare utilizations. We used the 2018 Area Deprivation Index (ADI) [38, 39], the 2015 Childhood Opportunity Index (COI) [40], and the 2018 Social Vulnerability Index (SVI) [41] to approximate the neighborhood characteristics and as proxies to patient socioeconomic status. Higher ADI rankings and SVI scores indicate more disadvantaged neighborhoods; but higher COI scores indicate more opportunities. All indices were transformed to have a range from 0 to 100.

### Analytic approach

We compared the differences in covariates and comorbidities between the C.L.I.M.B. and comparison groups in the pre- and post-periods using chi-square tests for categorical variables and t-tests for continuous variables. As in the tradition for propensity score (PS) analysis, we also presented the standardized differences (difference divided by the pooled standard deviation) between the two groups. When the absolute value of the standardized difference is greater than 0.1, it is indicative of non-negligible difference [42]. We estimated the DID effects using six statistical methods to triangulate evidence: 1) multivariable logistic regression adjustment (RA) controlling for comorbidities and covariates; 2) augmented inverse probability weighted (IPW) estimation [43] where covariates for the outcome and the PS models were selected using logistic lasso [44]; 3) IPW estimation where the PS was estimated using logistic regressions controlling for the same covariates in the RA model; 4) IPW-RA double robust method [45]; 5) bias-corrected single nearest neighbor matching method [46]; and 6) PS matching with a caliper 0.2. Each of these statistical methods has its own advantages and disadvantages. For example, the IPW and PS methods require the PS model to be correctly specified and the RA method assumes the outcome model is correctly specified. No one method clearly dominates others in terms of potential bias and relative efficiency. The 95% confidence intervals (CI) were estimated using the percentile-based bootstrap CI with 1,000 bootstrapped samples. All analyses were performed in Stata version 17 [47].

### Sensitivity analyses

We performed two sets of sensitivity analysis. First, we excluded 123 patients (236 admissions) who were in both pre- and post-periods, because the analyses may be contaminated by the correlations between observations for the same patients, especially when the patient was in different treatment groups across periods. Secondly, many RCTs include stringent inclusion/exclusion criteria. We applied some of the patient-selection criteria of the MOUD + A-CHESS trial [27] that can be defined using our data to assess the robustness of the main-analysis estimates in a selected sub-population who had no acute medical problems with immediate inpatient treatment needs, no history of psychotic disorders, and not pregnant.

## Results

A total of 2,320 unique patients with 2,443 inpatient detoxification admissions in the pre- and post-periods were included in the main analyses. Table 1 showed that in the pre-period, C.L.I.M.B. patients were more likely to be in the HMO plans, had more mood disorders, and diseases of the musculoskeletal system than patients in comparison facilities; however, in the post-period, C.L.I.M.B. patients had fewer other substance-related disorders, or diseases of the nervous or digestive systems than patients in the comparison facilities, mainly due to increased prevalence of these conditions in the comparison group. The largest and most significant differences between the groups were at the neighborhood level. C.L.I.M.B. patients were more likely to live in one the 100 largest metropolitan areas, and had lower ADI, higher COI, and lower SVI scores, i.e., they were from relatively more well-to-do neighborhoods.

**Table 1:** Demographic characteristics, medical claims six months prior to detoxification in the pre- and post-period.

	Pre-period			Post-period		
	Comparison	C.L.I.M.B.^d^	p-value ^c^	Comparison	C.L.I.M.B.^d^	p-value ^c^
	N = 1,385	N = 318		N = 545	N = 195	
Age category	N (%)	N (%)		N (%)	N (%)	
18–<25	407 (29.4)	88 (27.7)	0.636	154 (28.3)	53 (27.2)	0.47
25–<35	364 (26.3)	90 (28.3)		147 (27.0)	45 (23.1)	
35–<45	212 (15.3)	42 (13.2)		95 (17.4)	43 (22.1)	
45+	402 (29.0)	98 (30.8)		149 (27.3)	54 (27.7)	
Female	467 (33.7)	96 (30.2)	0.228	176 (32.3)	69 (35.4)	0.43
HMO	245 (17.7)	154 (48.4)	<0.001	57 (10.5)	87 (44.6)	<0.001
Comorbidity 6 months prior to index detoxification						
Had no claims	156 (11.3)	34 (10.7)	0.770	51 (9.4)	17 (8.7)	0.79
Had inpatient detoxification	78 (5.6)	18 (5.7)	0.984	48 (8.8)	15 (7.7)	0.63
Had emergency room visits	708 (51.1)	160 (50.3)	0.796	270 (49.5)	89 (45.6)	0.35
Had opioid use disorder diagnosis	677 (48.9)	160 (50.3)	0.645	295 (54.1)	102 (52.3)	0.66
Substance–related disorders ^a^	331 (23.9)	72 (22.6)	0.634	174 (31.9)	44 (22.6)	0.01
Mood disorders ^b^	550 (39.7)	148 (46.5)	0.026	236 (43.3)	84 (43.1)	0.96
Alcohol–related disorders	260 (18.8)	68 (21.4)	0.287	135 (24.8)	36 (18.5)	0.07
Anxiety/fear/trauma/stressor–related disorders	585 (42.2)	145 (45.6)	0.275	257 (47.2)	84 (43.1)	0.33
Suicidal ideation/attempt/intentional self–harm	130 (9.4)	31 (9.7)	0.842	45 (8.3)	12 (6.2)	0.35
Neoplasm	83 (6.0)	18 (5.7)	0.821	32 (5.9)	11 (5.6)	0.91
Endocrine, nutritional, and metabolic diseases	418 (30.2)	100 (31.4)	0.658	182 (33.4)	62 (31.8)	0.68
Diseases of the nervous system	569 (41.1)	120 (37.7)	0.273	229 (42.0)	66 (33.8)	0.05
Diseases of the circulatory system	448 (32.3)	92 (28.9)	0.238	185 (33.9)	57 (29.2)	0.23
Diseases of the respiratory system	381 (27.5)	83 (26.1)	0.611	155 (28.4)	58 (29.7)	0.73
Diseases of the digestive system	350 (25.3)	75 (23.6)	0.531	152 (27.9)	38 (19.5)	0.02
Diseases of the musculoskeletal system and connective tissue	653 (47.1)	130 (40.9)	0.043	238 (43.7)	78 (40.0)	0.37
Diseases of the genitourinary system	280 (20.2)	62 (19.5)	0.773	118 (21.7)	36 (18.5)	0.35
Injury, poisoning and certain other consequences of external causes	455 (32.9)	119 (37.4)	0.120	177 (32.5)	58 (29.7)	0.48
Live in one of the 100 largest metro areas	965 (69.7)	270 (84.9)	<0.001	377 (69.2)	169 (86.7)	<0.001
Neighborhood characteristics	Mean (SD) ^g^	Mean (SD) ^g^		Mean (SD) ^g^	Mean (SD) ^g^	
Mean ADI state rank ^e^	48.4 (21.6)	44.8 (23.9)	0.009	51.3 (22.3)	44.0 (24.4)	<0.001
Mean ADI national rank ^e^	59.8 (19.4)	57.1 (22.1)	0.033	61.1 (20.3)	55.6 (23.4)	<0.01
Mean childhood opportunity index	54.1 (20.8)	56.0 (23.4)	0.149	51.7 (21.6)	57.8 (24.2)	<0.01
Mean SVI socioeconomic score ^f^	44.9 (20.2)	42.0 (22.4)	0.022	47.0 (20.5)	39.5 (22.9)	<0.001
Mean SVI household/disability score	52.8 (18.2)	48.0 (18.7)	<0.001	54.1 (17.7)	46.0 (19.5)	<0.001
Mean SVI minority/language score	31.8 (17.7)	35.3 (16.8)	0.001	35.0 (19.0)	35.6 (17.3)	0.66
Mean SVI housing/transportation score	39.9 (14.6)	36.0 (14.7)	<0.001	42.3 (15.9)	34.1 (14.0)	<0.001

^a^ Including cannabis–, sedative–, stimulant–hallucinogen–or inhalant–related substances

^b^ Including depressive disorders, bipolar disorders, and other specified mood disorders

^c^ P–values are based on chi–square tests for categorical variables and t–tests for continuous variables

^d^ C.L.I.M.B. = Community-based Life-changing Individualized Medically assisted evidence-Based treatment

^e^ ADI = area deprivation index

^f^ SVI = social vulnerability index

^g^ SD = standard deviation

**Fig 2:**
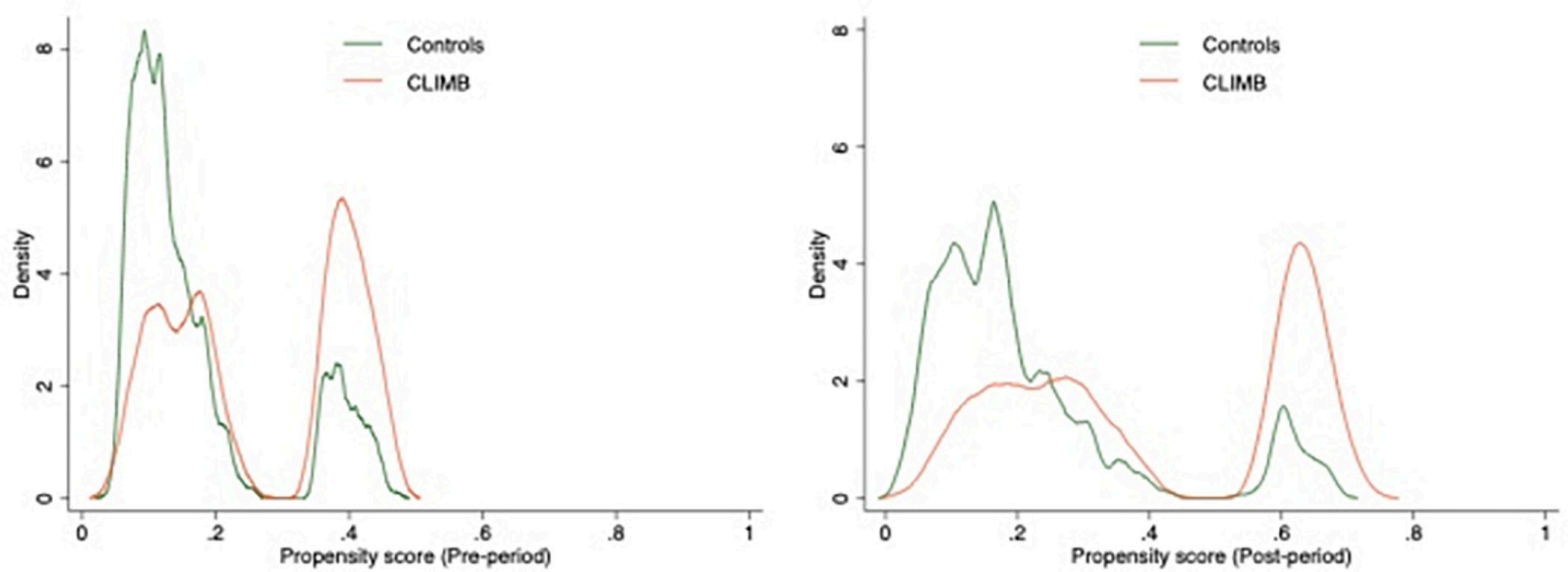
Propensity scores for participating in the pilot program in the pre- and post-periods. Using the plug-in lasso selected covariates from the covariates and the interactions between continuous and discrete covariates in Table 1. The selected variables for the pre-period propensity score are HMO×household/disability score, HMO×minority/language score, and HMO×housing/transportation score. The selected variables for the post-period propensity score are HMO×childhood opportunity index, HMO×minority/language score, and HMO×housing/transportation score.

Before using the PS for adjustments, the raw standardized differences (Table 2) showed consistent patterns as in Table 1. After weighting, the standardized differences were reduced to less than 0.1 for all except for 4 variables (mood disorder, disease of the musculoskeletal system and connective tissue, living in one of the 100 largest metropolitan areas, and mean SVI minority/language score, Table 2 column 2) in the pre-period; however, weighting did not improve balance in the post-period (17 variables had standardized difference greater than 0.1, Table 2 column 4). The residual imbalance was adjusted using these 4 and 17 variables in the nearest neighbor matching method. The few variables selected by the logistic lasso generated a bi-modal PS distribution (Fig 2) but there was good overlap between the PS of C.L.I.M.B. and comparison groups.

**Table 2:** Standardized difference (SD) between C.L.I.M.B.^a^ and comparison in the pre- and post-period respectively.

	Pre-period		Post-period	
	Raw SD ^d^	Weighted SD ^d^	Raw SD ^d^	Weighted SD ^d^
Age category				
18–<25	0.038	–0.078	0.024	0.019
25–<35	–0.046	0.025	0.089	–0.198
35–<45	0.059	–0.047	–0.119	0.105
45+	–0.039	0.090	–0.008	0.086
Female	0.075	–0.099	–0.066	0.168
HMO	–0.757	–0.040	–0.933	–0.060
Comorbidity 6 months prior to index detoxification				
Had no claims	0.018	0.021	0.022	–0.083
Had inpatient detoxification	–0.001	–0.030	0.040	–0.046
Had emergency room visits	0.016	–0.038	0.078	–0.070
Had opioid use disorder diagnosis	–0.029	0.025	0.037	–0.120
Substance–related disorders ^b^	0.030	–0.051	0.206	–0.161
Mood disorders ^c^	–0.139	0.143	0.005	–0.017
Alcohol–related disorders	–0.066	0.028	0.150	–0.129
Anxiety/fear/trauma/stressor–related disorders	–0.068	0.032	0.082	–0.078
Suicidal ideation/attempt/intentional self–harm	–0.012	–0.018	0.079	–0.187
Neoplasm	0.014	0.002	0.010	0.005
Endocrine, nutritional, and metabolic diseases	–0.028	0.062	0.034	–0.055
Diseases of the nervous system	0.068	–0.041	0.167	–0.223
Diseases of the circulatory system	0.073	–0.039	0.101	–0.135
Diseases of the respiratory system	0.032	–0.061	–0.029	–0.007
Diseases of the digestive system	0.039	–0.037	0.193	–0.192
Diseases of the musculoskeletal system and connective tissue	0.126	–0.103	0.074	–0.094
Diseases of the genitourinary system	0.018	–0.015	0.079	–0.026
Injury, poisoning and certain other consequences of external causes	–0.097	0.081	0.059	–0.063
Live in one of the 100 largest metro areas	–0.344	0.302	–0.404	0.274
Neighborhood characteristics				
Mean ADI state rank ^e^	0.163	0.022	0.317	–0.130
Mean ADI national rank ^e^	0.132	0.029	0.261	–0.138
Mean childhood opportunity index	–0.090	–0.078	–0.274	0.093
Mean SVI socioeconomic score ^f^	0.142	0.058	0.355	–0.143
Mean SVI household/disability score	0.265	0.019	0.443	–0.258
Mean SVI minority/language score	–0.202	0.168	–0.037	0.123
Mean SVI housing/transportation score	0.267	–0.068	0.529	–0.136

^a^ C.L.I.M.B. = Community-based Life-changing Individualized Medically assisted evidence-Based treatment

^b^ Including cannabis–, sedative–, stimulant–hallucinogen–or inhalant–related substances

^c^ Including depressive disorders, bipolar disorders, and other specified mood disorders

^d^ SD = standardized difference, comparison group minus treatment group divided by the pooled standard error

^e^ ADI: area deprivation index

^f^ SVI: social vulnerability index

All six methods (Table 3) gave similar estimated DRDs and RORs between groups over time. Before the pilot implementation, patients in C.L.I.M.B. facilities had statistically significantly higher 90-day readmission rates than patients in comparison facilities (17.0% vs. 10.2 to 11.7% estimated by various adjustment methods); however, after the pilot implementation, readmission rates decreased significantly in the C.L.I.M.B. patients to 12.3% whereas the adjusted rates in comparison facilities did not change significantly (varying from 11.8% to 15.3%). The DRDs and RORs were not statistically significant (adjusted DRDs ranged from 6 to 9 percentage points favoring the pilot group). Compared with that in the pre-period, patients’ profile changed a little in both groups, although the changes were not statistically significant in the C.L.I.M.B. group (S1 Table). The two sensitivity analyses (N = 2,197 observations in S2 Table and N = 2,121 observations in S3 Table) led to results qualitatively the same as the main analyses.

**Table 3:** Readmission rate in pre- and post-period and C.L.I.M.B.^a^ and comparison groups.

	Pre-period				Post-period				Treatment Effect			
	C.L.I.M.B. ^a^	Comparison	RD ^b^	OR ^c^	C.L.I.M.B. ^a^	Comparison	RD ^b^	OR^c^	DRD ^d^	95% CI ^f^	ROR ^e^	95% CI ^f^
Unadjusted	17.0	10.6	6.4	1.72	12.3	10.6	1.7	1.18	–4.6	[–11.8, 2.1]	0.68	[0.37, 1.26]
Logistic	17.0	10.2	6.8	1.85	12.3	11.8	0.5	1.05	–6.3	[–14.3, 1.4]	0.74	[0.39, 1.39]
AIPW Lasso ^g^	17.0	10.5	6.5	1.74	12.3	12.1	0.02	1.01	–5.9	[–14.4, 2.2]	0.61	[0.28, 1.30]
IPW ^h^	17.0	10.5	6.5	1.74	12.3	13.3	–1.0	0.92	–7.4	[–15.9, 0.3]	0.53	[0.25, 1.05]
IPWRA ^i^	17.0	10.5	6.5	1.74	12.3	12.9	–0.6	0.95	–7.1	[–14.7, 1.1]	0.54	[0.26, 1.09]
NNMATCH ^j^	17.0	11.7	5.3	1.55	12.3	14.8	–2.5	0.81	–7.8	[–17.5, 4.1]	0.52	[0.22, 1.70]
PSMATCH ^k^	17.0	11.0	6.0	1.66	12.3	15.3	–3.0	0.78	–9.0	[–19.0, 4.5]	0.47	[0.20, 1.70]

^a^ C.L.I.M.B. = Community-based Life-changing Individualized Medically assisted evidence-Based treatment

^b^ RD = risk difference

^c^ OR = odds ratio

^d^ DRD = difference of risk differences

^e^ ROR = ratio of odds ratios

^f^ CI = confidence interval. Percentile based on 1,000 bootstrapped samples.

^g^ AIPW = augmented inverse–probability weighting

^h^ IPW = inverse probability weighting

^I^ IPWRA = inverse probability weighted regression adjustment

^j^ NNMATCH = nearest–neighbor matching

^k^ PSMATCH = propensity–score matching

For the secondary outcomes of service utilization (Table 4), patients in comparison facilities experienced an increase in intensive outpatient care, partial hospitalization, and behavioral therapy within 30 days of discharge between pre- and post-periods, whereas patients in C.L.I.M.B. facilities did not. In both periods, patients in comparison facilities had higher partial hospitalization, lower outpatient services, and lower MOUD compared to patients in C.L.I.M.B. facilities; and in the post-period, patients in comparison facilities had higher behavioral therapy services than patients in C.L.I.M.B. facilities. When we adjusted for patient characteristics using logistic regressions, we found statistically significant difference in the RORs for behavioral therapy services received by patients in comparison vs. C.L.I.M.B. facilities, favoring the comparison facilities (Table 5).

**Table 4:** Proportions of patients receiving treatments within 30-day of the discharge date of the index inpatient detoxification.

	Pre-period		Post-period	
	Comparison	C.L.I.M.B. ^a^	Comparison	C.L.I.M.B. ^a^
	N = 1,385	N = 318	N = 545	N = 195
Intensive outpatient	238 (17.2%)	54 (17.0%)	121 (22.2%)	33 (16.9%)
Partial hospitalization	156 (11.3%)	11 (3.5%)	83 (15.2%)	4 (2.1%)
Domiciliary partial residential	36 (2.6%)	14 (4.4%)	21 (3.9%)	6 (3.1%)
Outpatient	477 (34.4%)	150 (47.2%)	180 (33.0%)	84 (43.1%)
Behavioral therapy	381 (27.5%)	84 (26.4%)	201 (36.9%)	37 (19.0%)
Medication for addiction treatment	269 (19.4%)	93 (29.2%)	102 (18.7%)	51 (26.2%)
Buprenorphine	147 (10.6%)	56 (17.6%)	46 (8.4%)	28 (14.4%)
Naltrexone oral or injectable	133 (9.6%)	42 (13.2%)	51 (9.4%)	24 (12.3%)
Injectable naltrexone	98 (7.1%)	23 (7.2%)	34 (6.2%)	15 (7.7%)
Oral naltrexone	89 (6.4%)	38 (11.9%)	31 (5.7%)	15 (7.7%)

^a^ C.L.I.M.B. = Community-based Life-changing Individualized Medically assisted evidence-Based treatment

**Table 5:** Difference-in-differences analyses of other treatments for opioid use disorder (OUD) 30 days after discharge using logistic regression adjusted by covariates.

	DRD^a^	ROR ^b^	95% CI ^c^ of ROR
Intensive outpatient	−4.0	0.75	[0.43, 1.29]
Partial hospitalization	−12.2	0.43	[0.13, 1.44]
Domiciliary partial residential	−2.9	0.40	[0.13, 1.27]
Outpatient	−1.5	0.87	[0.57, 1.33]
Behavioral therapy	−18.9	0.42	[0.25, 0.68]
Medication for OUD	−1.2	0.88	[0.54, 1.43]
Buprenorphine	−4.0	1.00	[0.54, 1.86]
Naltrexone oral or injectable	2.9	0.93	[0.49, 1.78]
Injectable naltrexone	2.7	1.27	[0.57, 2.84]
Oral naltrexone	−0.6	0.67	[0.31, 1.45]

^a^ DRD = difference of risk differences

^b^ ROR = ratio of odds ratios

^c^ CI = confidence interval. Percentile based on 1,000 bootstrapped samples.

## Discussion

The ASAM criteria advocate for individualized assessment-driven treatment and flexible use of services across a broad range of care, which can be offered by a single or multiple providers with “(1) seamless transfer between levels of care, (2) philosophical congruence among the various providers of care; and (3) timely arrival of the patient’s clinical record at the next provider” [48]. The C.L.I.M.B. program was designed using these principles. Although there was a significant decrease in 90-day readmission rates in the C.L.I.M.B. facilities from 17% to 12% from the pre- to post-period, compared with patients in comparison facilities the reductions were not statistically significant. There was no significant improvement in secondary outcomes, i.e., utilizations in lower LOC and MOUD in the C.L.I.M.B. facilities.

Compared with a small one-center pre-post study of buprenorphine treatment initiation in an intensive inpatient program, the C.L.I.M.B. program had lower outpatient utilization [49]. There is limited literature on the impact of community-based OUD chronic care models on reducing detoxification readmission after the index discharge. In a commercially insured population in the U.S., those entering care in an inpatient setting with only short-term inpatient stay without MOUD had an overdose rate of 4.3 per 100 person-years and an all-cause rehospitalization rate of 74.1 per 100 person-years [50]. A small retrospective study in an urban academic hospital found that in-hospital initiation of opioid agonist treatment through a hospital-based SUD consultation-liaison team did not reduce the 180-day all-cause rehospitalization compared with usual care [51]. However, a large RCT among eligible medical/surgical patients in the same setting with a more comprehensive patient-navigation service led to lower incidences of all-cause readmission in 30-, 90-, 180- and 365-days, but no significant difference in positive urine drug test [7]. These studies suggest treating OUD on an episodic basis without integrating all levels of care is unlikely to reduce readmission for detoxification.

Several reasons may explain our largely null findings. First, the pilot program was implemented in a period when many SUD facilities were undergoing changes in practice. While the pilot facilities had all ASAM LOC services, the comparison facilities may have varied cross-sectionally and over time. The National Survey of Substance Abuse Treatment Services (NSSAT) data showed that the proportion of SUD facilities in the U.S. that offered MOUD increased from 10% in 2007 to 36% in 2016 [52]. In a secret shopper audit study in 2019 Beetham et al. found 29% of residential treatment programs offer opioid agonist treatment [53]. Using the 2016 and 2019 NSSAT data, we found that the percent of facilities that offered a broad range of services had increased from 15% in 2016 to 21% in 2019. Hence, the comparison facilities may have experienced improvements in services in the study period. Although the rates in outpatient and MOUD treatments were higher in the C.L.I.M.B. facilities in both periods, the comparison facilities in our study had an increase in intensive outpatient care, partial hospitalization, and behavioral therapy within 30-day of discharge between the two periods, which might have explained part of the null findings. Through our connection with the providers in one large facility, we knew they initiated a similar program like the C.L.I.M.B. in the same period, and for that reason we excluded it from our post-pilot period.

Second, one of the key components of the pilot program was the integration of the smartphone app A-CHESS to the clinical practices. The C.L.I.M.B. therapist had some responsibility in tracking patients outside the therapy sessions and actively responding to “no show” appointments with identified contingencies of the treatment plan, such as contacting the pre-identified sober support persons to get the patient back on track. Unfortunately, few patients used A-CHESS after they signed up on the phone at discharge (confirmed by the A-CHESS data). Although access to a smartphone may be a limiting factor to patients with limited resources, providers’ feedback suggested that patients preferred other existing apps because the A-CHESS was not free and did not provide the feedbacks salient to the patients, e.g., money saved due to abstinence. It became clear that patient acceptance and provider integration of an app were needed for its successful utilization [54].

Third, the pilot program aimed to recruit 300 patients in the program, however, fewer than 200 patients enrolled. Due to funding termination of the pilot program, this evaluation was limited to the 16-month period after the program initiation. A post-hoc sample size analysis showed that in order to detect the observed effect size of the difference in readmission before and after the pilot with 80% power, we needed more than 5,000 patients in the two groups [55]. For this reason, findings from this paper should not be construed as evidence for denying services by insurers.

There are some strengths of our study. Although RCTs are deemed the gold standard to establish evidence of efficacy of a treatment, practitioners tend to find trial-tested treatments less effective in the real world. Community-based programs do not have strict inclusion/exclusion criteria as RCTs and use of a quasi-experimental design such as the DID method is a potentially valid approach to evaluating real world interventions.

Second, we used multiple statistical estimators to quantify the causal effect of interest and the estimates were largely consistent with each other, which was reassuring. Using a clearly defined causal effect of interest, i.e., the average treatment effect on the treated, which answered the specific question: “for patients treated in the pilot facilities, was the program a cause for the change in readmission rates?”, we did not lose sight of the goal of the evaluation.

Our study has several limitations. Foremost, although prevention of readmission was an important goal for the insurers, it may not have represented better outcomes for patients. Compared with measures of relapse in RCTs (e.g., 4 consecutive weeks of opioid use by urine toxicology or self-report, or 7 consecutive days of self-reported use in Lee et al. [56]), inpatient admissions do not capture all relapses. However, in the real world, health plans rely on these data to identify target population for quality improvement.

Secondly, a key untestable assumption for the DID methods is the parallel trend assumption (S2 Fig), i.e., the change in 90-day readmissions from pre- to post-periods in the comparison facilities is a good proxy for the counterfactual change in pilot facilities had there been no pilot intervention. In our data, the readmission rates in comparison facilities over the study period remained virtually unchanged. However, in the U.S. population from 2008 to 2016 there was significant decline in the rate of opioid-related discharges with detoxification services during hospitalization [2] which, although not a direct measure of readmission, presumably was indicative of declines in readmission for detoxification as well. As the treatment modality shifted toward MOUD delivered in an outpatient setting, it is possible that the change in comparison facilities did not reflect the counterfactual change that the pilot facilities would have experienced. Thus, our DID estimates may have over-estimated the true effect.

Thirdly, we may not have controlled for all relevant confounding factors in our analysis, including race/ethnicity. In addition, the disadvantaged neighborhood characteristics that we used to partially capture patients’ socioeconomic resources may have been misclassified. Based on our proxies, patients in the C.L.I.M.B. facilities lived in areas with higher psychosocial resources. However, higher proportions of patients in C.L.I.M.B. facilities were enrolled in HMO than PPO compared with patients in comparison facilities. It is generally true that HMO patients had lower socioeconomic resources, which was not reflected in our area-level measures. On the other hand, in both periods, comparison facilities had lower outpatient services and lower MOUD compared with pilot facilities, which is consistent with lower availability of treatment options. For the treatment to reach patients in remote rural areas or areas with few resources, better strategies need to be devised.

Fourthly, our patients were all privately insured and the pilot program that was approved by one insurer through less restriction in the length of detoxification stay and medication use may not generalize to other populations and settings. The findings that the C.L.I.M.B. patients mostly came from well-to-do neighborhood also suggested potential bias in the evaluation as the families of such participants often had more resources to get their relatives into other treatment facilities after leaving the pilot program, which might be related to low follow-up in the pilot facilities. Although the program was designed to reduce costs eventually through better engagement of patients in all levels of care, the reduction of inpatient readmission may not always coincide with better health outcomes.

Finally, many patients came to the pilot facilities from afar and after leaving the facilities they may not have completed the full spectrum of care in the pilot program or benefit from all the services offered due to different barriers. The Vermont Hub-and-Spoke model of care may help remove some of these barriers [57]. Initiating buprenorphine during a hospital admission [6] or emergency room visit [58] may improve treatment entry in the community. A patient navigation service that started in the hospital and continued for 3 months after discharge was related to a significant reduction in 30-day readmission [7]. The currently active (though not recruiting due to the COVID-19) large cluster randomized trial HEALing (Helping to End Addiction Long-term) Communities study will involve criminal justice settings, syringe service programs, mental health/addiction treatment programs, primary care, other general medical and behavioral health settings, and recovery programs to implement a broad array of evidence-based interventions through a community-driven process [59]. Finding factors that improve treatment engagement and retention is an important next step in the design for effective intervention in the future.

### Clinical implication

The reason that OUD needs to be treated as a chronic condition is due to the complexity of the disorder. Individuals with OUD cannot during the withdrawal (detoxification) process learn the tasks needed to surmount the biologic and psychological aspects of the illness. The treatment to sustain wellness and provide education about the illness requires continued and frequent engagement for a sustained period. Being able to understand what has and will likely occur during the recovery process through thoughtful development of contingency planning is essential. Having insight into the psychological issues that may predate the actual dependence on the substance needs significant work at first and then continuous engagement for very long periods of time as well. These are all characteristics of chronic disease and chronic disease management principles. We found that even though health plans used alternative utilization management process that allowed more time to be spent in the residential/domiciliary and other mid-levels of care and providers agreed to use codified treatment plans, additional strategies need to be developed to encourage engagement and reduce readmission related to relapse.

## Conclusions

Our study used a quasi-experimental design to evaluate the impact of a community-based program on reducing inpatient detoxification readmission. The pilot was designed based on a chronic care model for OUD in the community [17]. Although there was a reduction in readmission in the pilot facilities between the two periods, the utilization of lower level of care services remained low. Even though providers in the pilot OUD treatment facilities actively worked with health plans to standardize care for patients with OUD, more strategies are needed to improve treatment engagement and retention after an inpatient detoxification.

## Supporting information

S1 Fig Opioid chronic condition clinical pathway in weeks (w) and months (m). (DOCX)

S2 Fig Causal effects in a difference–in–differences analysis. Solid black lines for observed data, black dashed line for the estimated potential outcome for the pilot group and the red dotted line for bias using a pre-post design with only the pilot group data.
(DOCX)

S1 Table Demographic characteristics, medical claims six months prior to the index detoxification between the pre- and post-period for each treatment group. (DOCX)

S2 Table Sensitivity analysis of 90-day readmission rate in pre- and post-period and C.L.I.M.B. and comparison groups, excluding patients belonging to two periods. (DOCX)

S3 Table Sensitivity analysis of 90-day readmission rate in pre- and post-period and C.L.I.M.B. and comparison groups, using some exclusion criteria of the MOUD + A-CHESS trial. (DOCX)

S1 Appendix C.L.I.M.B. pilot protocol. (DOCX)

## List of abbreviations

C.L.I.M.B.: community-based life-changing individualized medically assisted evidence-based treatment
OUD: opioid use disorder
DID: difference-in-differences
ASAM: American Society of Addiction Medicine
BCN: Blue Care Network
BCBSM: Blue Cross Blue Shield of Michigan
DRD: difference of risk differences
OR: odds ratio
ROR: ratio of odds ratios
PPO: preferred provider organization
HMO: health maintenance organization
ADI: area deprivation index
COI: childhood opportunity index
SVI: social vulnerability index
RA: regression adjustment
IPW: inverse probability weighted
PS: propensity scores
CI: confidence interval
RCT: randomized controlled trial
MOUD: medication for opioid use disorder
A-CHESS: Addiction-Center for Health Enablement Support System
LOC: level of care
SUD: substance use disorder
ICD-10-CM: International Classification of Diseases, 10^th^ version, Clinical Modification
