# Supplementary material for: Prevention of 90-day inpatient detoxification readmission for opioid use disorder by a community-based life-changing individualized medically assisted evidence-based treatment (C.L.I.M.B.) program: A quasi-experimental study
Source: PLoS One. 2022 Dec 15;17(12):e0278208. doi: 10.1371/journal.pone.0278208 (PMC9754176; doi:10.1371/journal.pone.0278208)
Supplement: S1 Fig — (DOCX) [file pone.0278208.s001.docx]

**Figure S1. Opioid Chronic Condition Clinical Pathway in weeks (w) and months (m)**

|  |  |  |  |  |  |  |  |  |  |  |
| --- | --- | --- | --- | --- | --- | --- | --- | --- | --- | --- |

1w 2w 3w 4w 5w 6w 7w 2 m 4m 8m 10m 12m+

Detoxification (1-3 days): ASAM 3.5-4.0, Day 1-6

Domiciliary LOC (Inpatient/Residential/DPHP/DIOP) = (7-14 days): ASAM 3.1-3.5, D1-6

Intensive Outpatient Program (IOP) = (4-8+ weeks): ASAM 2.1, D1-6 to 2.5, D2-6

Outpatient Services = (12+ months; may be more

intensive sessions earlier on 1-3x/week

Ind./Group/Family) as needed. ASAM 1.0, D1-6
