# Supplementary material for: Prevention of 90-day inpatient detoxification readmission for opioid use disorder by a community-based life-changing individualized medically assisted evidence-based treatment (C.L.I.M.B.) program: A quasi-experimental study
Source: PLoS One. 2022 Dec 15;17(12):e0278208. doi: 10.1371/journal.pone.0278208 (PMC9754176; doi:10.1371/journal.pone.0278208)

**Figure S2. Causal effects in a difference–in–differences analysis.** Solid black lines for observed data, black dashed line for the estimated potential outcome for the pilot group and the red dotted line for bias using a pre-post design with only the pilot group data.


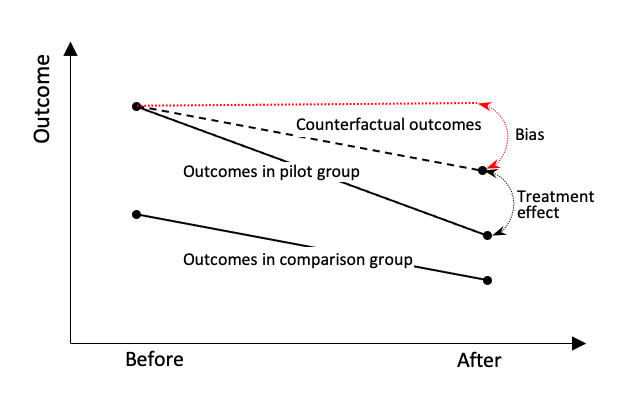

Supplement: S2 Fig — Solid black lines for observed data, black dashed line for the estimated potential outcome for the pilot group and the red dotted line for bias using a pre-post design with only the pilot group data. (DOCX) [file pone.0278208.s002.docx]
