# Supplementary material for: Prevention of 90-day inpatient detoxification readmission for opioid use disorder by a community-based life-changing individualized medically assisted evidence-based treatment (C.L.I.M.B.) program: A quasi-experimental study
Source: PLoS One. 2022 Dec 15;17(12):e0278208. doi: 10.1371/journal.pone.0278208 (PMC9754176; doi:10.1371/journal.pone.0278208)
Supplement: S1 Table — (DOCX) [file pone.0278208.s003.docx]

**Table S1. Demographic characteristics, medical claims six months prior to the index detoxification between the pre- and post-period for each treatment group**

|  | **C.L.I.M.B.** ^a^ | | | | **Comparison** | | | |
| --- | --- | --- | --- | --- | --- | --- | --- | --- |
|  | **Pre-period** | **Post-period** | **p-value** ^b^ | **Standardized difference** | **Pre-period** | **Post-period** | **p-value** ^b^ | **Standardized difference** |
|  | N=318 | N=195 |  |  | N=1,358 | N=545 |  |  |
| Age category | N (%) | N (%) |  |  | N (%) | N (%) |  |  |
| 18–<25 | 88 (27.7) | 53 (27.2) | 0.061 | –0.011 | 407 (29.4) | 154 (28.3) | 0.628 | –0.025 |
| 25–<35 | 90 (28.3) | 45 (23.1) |  | –0.119 | 364 (26.3) | 147 (27.0) |  | 0.016 |
| 35–<45 | 42 (13.2) | 43 (22.1) |  | 0.239 | 212 (15.3) | 95 (17.4) |  | 0.058 |
| 45+ | 98 (30.8) | 54 (27.7) |  | –0.068 | 402 (29.0) | 149 (27.3) |  | –0.037 |
| Female | 96 (30.2) | 69 (35.4) | 0.221 | 0.111 | 467 (33.7) | 176 (32.3) | 0.550 | –0.030 |
| HMO | 154 (48.4) | 87 (44.6) | 0.401 | –0.076 | 245 (17.7) | 57 (10.5) | <0.001 | –0.200 |
| Comorbidity 6 months prior to index detoxification |  |  |  |  |  |  |  |  |
| Had no claims | 34 (10.7) | 17 (8.7) | 0.468 | –0.066 | 156 (11.3) | 51 (9.4) | 0.223 | –0.062 |
| had detoxification | 18 (5.7) | 15 (7.7) | 0.363 | 0.083 | 78 (5.6) | 48 (8.8) | 0.011 | 0.129 |
| Had emergency room visits | 160 (50.3) | 89 (45.6) | 0.304 | –0.094 | 708 (51.1) | 270 (49.5) | 0.533 | –0.032 |
| Had opioid use disorder diagnosis | 160 (50.3) | 102 (52.3) | 0.661 | 0.040 | 677 (48.9) | 295 (54.1) | 0.038 | 0.105 |
| Substance–related disorders ^c^ | 72 (22.6) | 44 (22.6) | 0.984 | –0.002 | 331 (23.9) | 174 (31.9) | <0.001 | 0.183 |
| Mood disorders ^d^ | 148 (46.5) | 84 (43.1) | 0.444 | –0.070 | 550 (39.7) | 236 (43.3) | 0.148 | 0.073 |
| Alcohol–related disorders | 68 (21.4) | 36 (18.5) | 0.424 | –0.073 | 260 (18.8) | 135 (24.8) | 0.003 | 0.149 |
| Anxiety/fear/trauma/stressor–related disorders | 145 (45.6) | 84 (43.1) | 0.577 | –0.051 | 585 (42.2) | 257 (47.2) | 0.050 | 0.099 |
| Suicidal ideation/attempt/intentional self–harm | 31 (9.7) | 12 (6.2) | 0.154 | –0.130 | 130 (9.4) | 45 (8.3) | 0.437 | –0.039 |
| Neoplasm | 18 (5.7) | 11 (5.6) | 0.993 | –0.001 | 83 (6.0) | 32 (5.9) | 0.919 | –0.005 |
| Endocrine, nutritional, and metabolic diseases | 100 (31.4) | 62 (31.8) | 0.934 | 0.007 | 418 (30.2) | 182 (33.4) | 0.170 | 0.069 |
| Diseases of the nervous system | 120 (37.7) | 66 (33.8) | 0.374 | –0.081 | 569 (41.1) | 229 (42.0) | 0.707 | 0.019 |
| Diseases of the circulatory system | 92 (28.9) | 57 (29.2) | 0.942 | 0.007 | 448 (32.3) | 185 (33.9) | 0.501 | 0.034 |
| Diseases of the respiratory system | 83 (26.1) | 58 (29.7) | 0.370 | 0.082 | 381 (27.5) | 155 (28.4) | 0.681 | 0.021 |
| Diseases of the digestive system | 75 (23.6) | 38 (19.5) | 0.277 | –0.099 | 350 (25.3) | 152 (27.9) | 0.238 | 0.060 |
| Diseases of the musculoskeletal system and connective tissue | 130 (40.9) | 78 (40.0) | 0.844 | –0.018 | 653 (47.1) | 238 (43.7) | 0.168 | –0.070 |
| Diseases of the genitourinary system | 62 (19.5) | 36 (18.5) | 0.772 | –0.026 | 280 (20.2) | 118 (21.7) | 0.483 | 0.035 |
| Injury, poisoning and certain other consequences of external causes | 119 (37.4) | 58 (29.7) | 0.076 | –0.162 | 455 (32.9) | 177 (32.5) | 0.874 | –0.008 |
| Live in one of the 100 largest metro areas | 270 (84.9) | 169 (86.7) | 0.582 | 0.050 | 965 (69.7) | 377 (69.2) | 0.830 | –0.011 |
| Neighborhood characteristics | Mean (SD) ^g^ | Mean (SD) ^g^ |  |  | Mean (SD) ^g^ | Mean (SD) ^g^ |  |  |
| Mean ADI state rank ^e^ | 44.8 (23.9) | 44.0 (24.4) | 0.734 | –0.031 | 48.4 (21.6) | 51.3 (22.3) | 0.009 | 0.133 |
| Mean ADI national rank ^e^ | 57.1 (22.1) | 55.6 (23.4) | 0.459 | –0.067 | 59.8 (19.4) | 61.1 (20.3) | 0.175 | 0.069 |
| Mean childhood opportunity index | 56.0 (23.4) | 57.8 (24.2) | 0.407 | 0.076 | 54.1 (20.8) | 51.7 (21.6) | 0.024 | –0.114 |
| Mean SVI socioeconomic score ^f^ | 42.0 (22.4) | 39.5 (22.9) | 0.232 | –0.109 | 44.9 (20.2) | 47.0 (20.5) | 0.040 | 0.104 |
| Mean SVI household/disability score ^f^ | 48.0 (18.7) | 46.0 (19.5) | 0.252 | –0.104 | 52.8 (18.2) | 54.1 (17.7) | 0.180 | 0.068 |
| Mean SVI minority/language score ^f^ | 35.3 (16.8) | 35.6 (17.3) | 0.832 | 0.019 | 31.8 (17.7) | 35.0 (19.0) | 0.001 | 0.176 |
| Mean SVI housing/transportation score ^f^ | 36.0 (14.7) | 34.1 (14.0) | 0.160 | –0.128 | 39.9 (14.6) | 42.3 (15.9) | 0.002 | 0.161 |

^a^ C.L.I.M.B. = Community-based Life-changing Individualized Medically assisted evidence-Based treatment

^b^ p–values are based on chi-square tests for categorical variables and t-tests for continuous variables

^c^ Including cannabis-, sedative-, stimulant-, hallucinogen- or inhalant-related substances

^d^ Including depressive disorders, bipolar disorders, and other specified mood disorders

^e^ ADI = area deprivation index

^f^ SVI = social vulnerability index

^g^ SD = standard deviation

Table S1 shows the changes in patient profiles from the pre- to post-period in the C.L.I.M.B. group and the comparison group respectively. We can see in the C.L.I.M.B. group there were some increases in the proportions of patients 35-<45 years of age, female, but decreases in suicidal ideation, injury, poisoning, and other consequences of external causes, and better neighborhood characteristics. On the other hand, in the comparison group, there were decreases in the proportion of HMO patients, but increases in patients with history of OUD, substance use and alcohol-related disorders, and worsening of neighborhood characteristics. Due to smaller sample size in the C.L.I.M.B. group, none of the changes in patient profiles between periods are statistically significant at the 0.05 level, whereas the changes of the aforementioned characteristics in the comparison group are statistically significant at the 0.05 level.
