# Supplementary material for: Prevention of 90-day inpatient detoxification readmission for opioid use disorder by a community-based life-changing individualized medically assisted evidence-based treatment (C.L.I.M.B.) program: A quasi-experimental study
Source: PLoS One. 2022 Dec 15;17(12):e0278208. doi: 10.1371/journal.pone.0278208 (PMC9754176; doi:10.1371/journal.pone.0278208)
Supplement: S2 Table — (DOCX) [file pone.0278208.s004.docx]

**Table S2. Sensitivity analysis of 90-day readmission rate in pre- and post-period and C.L.I.M.B. ^a^ and comparison groups, excluding patients belonging to two periods**

|  | **Pre-period** | | | | **Post-period** | | | | **Treatment Effect** | | | | |
| --- | --- | --- | --- | --- | --- | --- | --- | --- | --- | --- | --- | --- | --- |
|  | C.L.I.M.B. | Comparison | RD ^b^ | OR ^c^ | C.L.I.M.B. | Comparison | RD ^b^ | OR ^c^ | DRD ^d^ | 95% CI ^f^ | ROR ^e^ | 95% CI ^f^ |  |
| Unadjusted | 14.7 | 9.5 | 5.3 | 1.65 | 9.4 | 7.7 | 1.7 | 1.25 | –3.5 | [–10.1, 3.5] | 0.76 | [0.36, 1.58] |  |
| Adjusted | 14.7 | 9.5 | 5.2 | 1.68 | 9.4 | 7.9 | 1.5 | 1.23 | –3.7 | [–11.9, 4.3] | 0.80 | [0.38, 1.71] |  |
| AIPW Lasso ^g^ | 14.7 | 9.9 | 4.8 | 1.57 | 9.4 | 8.7 | 0.1 | 1.09 | –4.1 | [–12.6, 3.3] | 0.69 | [0.26, 1.60] |  |
| IPW ^h^ | 14.7 | 9.3 | 5.4 | 1.69 | 9.4 | 8.4 | 0.1 | 1.13 | –4.5 | [–11.6, 2.5] | 0.67 | [0.28, 1.46] |  |
| IPWRA ^i^ | 14.7 | 9.3 | 5.4 | 1.68 | 9.4 | 8.2 | 1.2 | 1.16 | –4.2 | [–11.5, 2.5] | 0.69 | [0.28, 1.49] |  |
| NNMATCH ^j^ | 14.7 | 9.0 | 5.7 | 1.74 | 9.4 | 15.8 | –6.4 | 0.55 | –12.1 | [–20.9, 1.8] | 0.32 | [0.14, 1.40] |  |
| PSMATCH ^k^ | 14.7 | 10.4 | 4.3 | 1.49 | 9.4 | 11.2 | –1.8 | 0.82 | –6.1 | [–18.1, 3.9] | 0.55 | [0.16, 1.82] |  |

^a^ C.L.I.M.B. = Community-based Life-changing Individualized Medically assisted evidence-Based treatment

^b^ RD = risk difference

^c^ OR = odds ratio

^d^ DRD = difference of risk differences

^e^ ROR = ratio of odds ratios

^f^ CI = confidence interval. Percentile-based CI with 1,000 bootstrap samples

^g^ AIPW = augmented inverse probability weighting

^h^ IPW = inverse probability weighting

^i^ IPWRA = inverse probability weighted regression adjustment

^j^ NNMATCH = nearest neighbor matching

^k^ PSMATCH = propensity score matching within 0.2 caliper

Sensitivity Analysis 1 (Table S2) excludes patients belonging to two periods. The main analysis uses 2,320 unique patients and 2,443 observations. This sensitivity analysis uses data of 2,197 unique patients and 2,197 observations.
