# Supplementary material for: Prevention of 90-day inpatient detoxification readmission for opioid use disorder by a community-based life-changing individualized medically assisted evidence-based treatment (C.L.I.M.B.) program: A quasi-experimental study
Source: PLoS One. 2022 Dec 15;17(12):e0278208. doi: 10.1371/journal.pone.0278208 (PMC9754176; doi:10.1371/journal.pone.0278208)
Supplement: S3 Table — (DOCX) [file pone.0278208.s005.docx]

**Table S3. Sensitivity analysis of 90-day readmission rate in pre- and post-period and C.L.I.M.B. ^a^ and comparison groups, using some exclusion criteria of the MOUD + A-CHESS trial. ^m^**

|  | **Pre-period** | | | | **Post-period** | | | | **Treatment Effect** | | | | |
| --- | --- | --- | --- | --- | --- | --- | --- | --- | --- | --- | --- | --- | --- |
|  | C.L.I.M.B. | Comparison | RD ^b^ | OR ^c^ | C.L.I.M.B. | Comparison | RD ^b^ | OR ^c^ | DRD ^d^ | 95% CI ^f^ | ROR ^e^ | 95% CI ^f^ |  |
| Unadjusted | 16.8 | 10.3 | 6.5 | 1.76 | 12.6 | 10.7 | 1.9 | 1.21 | –4.6 | [–11.6, 3.0] | 0.69 | [0.36, 1.30] |  |
| Adjusted | 16.8 | 10.5 | 6.4 | 1.77 | 12.6 | 12.7 | –0.1 | 0.99 | –6.8 | [–15.5, 2.7] | 0.73 | [0.38, 1.37] |  |
| AIPW Lasso ^g^ | 16.8 | 11.0 | 5.9 | 1.64 | 12.6 | 11.5 | 1.1 | 1.11 | –4.8 | [–13.7, 3.3] | 0.68 | [0.30, 1.39] |  |
| IPW ^h^ | 16.8 | 10.4 | 6.4 | 1.74 | 12.6 | 14.6 | –1.9 | 0.85 | –8.3 | [–17.1, 0.2] | 0.49 | [0.24, 1.01] |  |
| IPWRA ^i^ | 16.8 | 10.4 | 6.4 | 1.74 | 12.6 | 13.9 | –1.3 | 0.90 | –7.7 | [–16.1, 0.6] | 0.52 | [0.26, 1.03] |  |
| NNMATCH ^j^ | 16.8 | 9.9 | 6.9 | 1.83 | 12.6 | 18.0 | –5.4 | 0.66 | –12.3 | [–22.2, 1.7] | 0.36 | [0.16, 1.23] |  |
| PSMATCH ^k^ | 16.8 | 9.4 | 7.5 | 1.96 | 12.6 | 13.9 | –1.2 | 0.90 | –8.7 | [–21.2, 3.2] | 0.46 | [0.18,1.40] |  |

^a^ C.L.I.M.B. = Community-based Life-changing Individualized Medically assisted evidence-Based treatment

^b^ RD = risk difference

^c^ OR = odds ratio

^d^ DRD = difference of risk differences

^e^ ROR = ratio of odds ratios

^f^ CI = confidence interval. Percentile-based CI with 1,000 bootstrap samples

^g^ AIPW = augmented inverse probability weighting

^h^ IPW = inverse probability weighting

^i^ IPWRA = inverse probability weighted regression adjustment

^j^ NNMATCH = nearest neighbor matching

^k^ PSMATCH = propensity score matching within 0.2 caliper

^m^ MAT+A-CHESS = Medication assisted treatment app for Addiction support at the Center for Health Enablement Support System

Sensitivity Analysis 2 (Table S3) excludes patients using some of the criteria in the MOUD+A-CHESS randomized controlled trial, excluding patients with acute medical problems with immediate inpatient treatment needs (acute posthemorrhagic anemia, rheumatic heart disease, acute myocardial infarction or complication with myocardial infarction, acute pulmonary embolism, hemorrhagic cerebrovascular disease, acute phlebitis; thrombophlebitis and thromboembolism, renal failure, acute lymphoblastic or myeloid leukemia, or acute bronchitis), patients with history of psychotic problems, or pregnant. This sensitivity analysis uses data of 2,022 unique patients and 2,121 observations.
