# Supplementary material for: Prevention of 90-day inpatient detoxification readmission for opioid use disorder by a community-based life-changing individualized medically assisted evidence-based treatment (C.L.I.M.B.) program: A quasi-experimental study
Source: PLoS One. 2022 Dec 15;17(12):e0278208. doi: 10.1371/journal.pone.0278208 (PMC9754176; doi:10.1371/journal.pone.0278208)
Supplement: S1 Appendix — (DOCX) [file pone.0278208.s006.docx]

**Appendix for “Prevention of 90-day inpatient detoxification readmission for opioid use disorder by a community-based life-changing individualized medically assisted evidence-based treatment (C.L.I.M.B.) program: a quasi-experimental study”**

**C.L.I.M.B. pilot protocol**

Working with our provider partners we codified an operating procedure that was based on currently available evidence-based treatment and ideally will become a new standard of care for members. It is an alternative utilization management process to facilitate treatment that takes into consideration the chronicity of the illness. This means that more time may be spent in the residential/domiciliary portion of treatment while mid-level and outpatient services will be extended even further out. This allows for continued treatment of withdrawal potential and biomedical conditions in a lower level of care (LOC) and presents opportunity to reduce the time in the sub-acute detoxification phase.

The sub-acute detoxification phase (ASAM LOC 3.3 or higher) should not change and, if anything, could be somewhat shorter with continuing withdrawal addressed in the domiciliary LOC. Because of the “genetic memory” that the body has with opioids, patients are a high risk for rapidly becoming dependent and or having a fatal overdose should they relapse.

The use of the domiciliary/residential LOC (ASAM LOC 3.0-3.1) provides a safe and sober living environment to reduce this risk after detoxification. Other objectives should include planning for a suitable recovery environment and medication for opioid use disorder (MOUD), if appropriate. Evidence-based pharmacotherapy options such as Buprenorphine and Naltrexone (po or im) may be considered. Antabuse may be used for some members with co-occurring alcohol use disorders. Anticonvulsants, antidepressants, and mood stabilizers may be instituted, if appropriate, for emotional and behavioral conditions. Education regarding the A-CHESS tool would be provided.

Following the domiciliary LOC, the partial hospitalization program/intensive outpatient program (PHP/IOP, ASAM LOC 2.0-2.5) would continue the work started in the domiciliary LOC. The member could be living at home or in a half-way or 3/4 housing situation, ideally be back to work or school and simultaneously attending 12 step programming. They would be using the A-CHESS app and having routine drug testing with weekly reporting to the therapist. The PHP/IOP service would continue for 12-24 sessions on the average depending on the needs of the member. While this may be an average, some members may need longer or shorter times in this LOC.

Once the ASAM LOC 2.0 is completed, the member will transition to outpatient care (ASAM 1.0). They will still be involved in routine drug testing, A-CHESS app use, medication for addiction treatment, 12 step programming for the remainder of at least a year of treatment overall. Clinical variation will occur between individuals but generally 80% of members would utilize this protocol.

It’s recognized that relapses will occur. Having the master treatment plan clearly identified before transition to another LOC would be imperative to coordinate the expected response to the relapse. Coordination with other providers that may be providing the next LOC is critical. Risk would have to be assessed to determine the appropriate intervention such as: returning to a higher LOC to stabilize, increasing outpatient sessions over a short period of time, or more frequent use of the A-CHESS app to get back on track.

The timeline is shown in Fig S1.

*Critical tasks to accomplish in each LOC*

*Detoxification:* 1-3 days in LOC appropriate for risk of withdrawal potential, biomedical conditions and complications and comorbid emotional and behavioral conditions. For opioids, it is extremely rare that there is a truly life-threatening issue that needs 24/7 medical treatment. Members can be very uncomfortable with the withdrawal symptoms, but if common supportive measures and interventions are prescribed (not just medications) members can be kept safe and more comfortable. Using medications such as Buprenorphine in the withdrawal process can “jump start’ the MOUD process itself and shorten the time required at this LOC while simultaneously addressing symptom relief. The critical element of this LOC is to stabilize the members physical symptoms of withdrawal and move on to the Domiciliary LOC where they can initiate the other critical items that need to occur in their treatment journey.

*Domiciliary/Residential LOC:* 7-14 days in ASAM 3.1 or less. This LOC goes by many acronyms, but the overall intervention is to provide a safe structured recovery environment with lesser temptation to relapse and have access to medical attention if required to address stabilizing withdrawal symptoms. Critical items that will need to be addressed beyond biomedical and withdrawal conditions are education about the disease, assessing motivation to change and tools to reduce relapse potential. Examples would include initiation or plan for continuation of medication where appropriate, education about the social programs including the use of technology (A-CHESS) in the recovery. These critical milestones need to occur in a structured method like a curriculum which is based on mastery of the critical tasks at each step to graduate to the next one.

*PHP/IOP LOC:* 12-24 Sessions. This LOC is the first time the members get to practice what they have learned and committed to. Education and treatment components occur while allowing newly learned skills to be practiced in the real-world environment with continued support and structure of the programming. Addressing critical items at roughly 2-week intervals would seem a good pace towards completion of this LOC but with a 2-month average LOS. Items in this category would be identification of sober supports and their reliability, addressing family support or lack thereof and how to address this through concrete living arrangement decisions in the short and possibly longer term. The next two-week interval would likely focus on work or education relationships along with a deeper dive into triggers for relapse. Stabilization using medication, review of drug testing and missed appointments, adherence to A-CHESS, and follow-up with therapist and psychiatry appointments Development of the curriculum jointly will be a process but as you can see each step needs to build on the prior one and the step cannot go forth without mastery of the key areas of focus.

*Outpatient LOC:* 12 months or more on average. This transition will have phases as well. It is of a longer-term duration but critical items will need to be addressed each month or two. Developing benchmarks and celebration of achievement with the member will need to be built into the programming. The therapy relationship may also be different than treatment typically has been. Interactions will need to monitor the member through A-CHESS, have routine contact with the sponsor (sober coach) and likely sober supports. This will take some aspect of coordination of care with the treatment team that involves all these extraordinary treatment contacts and use of motivational interviewing and behavioral activation skills to support continued recovery.

*Critical Elements of Each Level:*

Detoxification: Standard medical detoxification as needed for member.

Domiciliary/Residential: First 2-4 days, continue taper of medications started in detoxification. Complete psychosocial and medical exam. Provide supportive care for PAWS, start psychoeducation, family support session. Engage in motivational interviewing to assess for commitment/readiness for change. Begin considering discharge planning, housing considerations for next level. Initiate pharmacotherapy as appropriate for opioids, polysubstance use, alcohol, or chronic pain. Initiate and educate regarding A-CHESS mobile app.

PHP/IOP: Occurs 3-4x/week, from home, transitional housing. Includes drug testing and 5-7x/week 12-step recovery programming. Psychiatric follow-up for mental health issues, possible referral to Adult Intensive Services if dual diagnosis. Family sessions, MOUD to continue, continue A-CHESS app.

Outpatient: Individual and/or group therapy 1-3x/week, family therapy, continued 12-step support, drug testing 1-3x/week. Evaluate progress, continue A-CHESS participation, continue psychiatry follow-up and MOUD. Outpatient treatment intensity is driven by individual treatment planning and needs.

*Practical implications:*

This pilot will have some changes not only in the treatment received by the member but how that treatment is provided. The therapist has some responsibility in tracking the member outside the therapy session. Especially in the outpatient component monitoring drug testing and the results, monitoring A-CHESS adherence and participation, implementing consequences or contingencies of treatment plan violations, actively responding to “no show” appointments with identified contingencies of the treatment plan and interacting with sober supports.

The plan will also have different responsibilities. BCN will continue to monitor utilization and manage the care utilizing InterQual criteria which includes ASAM components. The result will be that more of these cases will go to the medical advisor for review and adjudication. This protocol does not do away with the utilization management process but should assist expectations on both partners involvement. The purpose is not to increase utilization thinking that this alone will solve the problem for the member but to utilize the interventions in the members best interest while being mindful of the proposed cost of services verses the benefit that can be derived. As an example, to illustrate this point the member staying in an inpatient level of detoxification for one more day can purchase approximately10 outpatient visits and extend the time in treatment for 2.5 months.

Finally, patients with opioid dependence who received medication for this disorder had lower hospital utilization and total costs than patients who did not receive pharmacologic therapy, illustrating the importance placed on MOUD in this pilot.

The timeline of the intervention is shown in Figure S1.
